# Implementation of the COVID-19 antiviral therapy Nirmatrelvir/Ritonavir (Paxlovid^TM^) across Canada in 2022: A qualitative analysis of key facilitating factors and challenges

**DOI:** 10.14745/ccdr.v51i08a03

**Published:** 2025-08-28

**Authors:** Aklile Workneh, Camilia Thieba, Nadine Sicard

**Affiliations:** 1 Public Health Agency of Canada, Ottawa, ON; 2 Public Health Agency of Canada, Montréal, QC

**Keywords:** COVID-19, therapeutics, nirmatrelvir/ritonavir, implementation, Canada

## Abstract

**Background:**

The COVID-19 antiviral Nirmatrelvir/Ritonavir (Paxlovid^TM^, N/R) was approved for use in Canada in January 2022, with the Government of Canada assuming a procurement role and provinces, territories, and federal departments implementing usage within their respective healthcare systems. The objective of this analysis is to describe how N/R was implemented across various jurisdictions in the first six months after it was available for use and identify promising implementation practices.

**Methods:**

Fourteen semi-structured discussions in small group settings were conducted with jurisdictional representatives involved in the implementation of N/R. A descriptive analysis of the eligibility criteria and service delivery model was conducted. A thematic analysis using the Consolidated Framework for Implementation Research and cluster analysis of the codes were then undertaken on NVivo 12 to identify key themes.

**Results:**

Overall, the eligibility criteria were similar across jurisdictions, and three types of service delivery models were identified. Ten main themes emerged as facilitators and eight as challenges to the implementation. Partnership, collaboration, communication and flexibility were among the facilitators identified, while the complexity of the intervention (e.g., drug-drug interactions), perceived evidence gaps in effectiveness by prescribers, and resource limitations were identified as key implementation challenges.

**Conclusion:**

While there were jurisdictional variations in the implementation of N/R, communication and collaboration, and the availability of rapid testing for COVID-19 emerged as key facilitators. Drug-drug interactions, resource pressures and limited evidence were some of the key challenges. Overall, these facilitators and challenges were similar across jurisdictions and may help inform future therapeutic implementation plans for pandemic preparedness.

## Introduction

Nirmatrelvir/Ritonavir (Paxlovid^TM^, N/R) is an oral antiviral for treatment of SARS-CoV-2 in adults with mild to moderate symptoms at high-risk of progressing to severe disease or death. On January 17, 2022, Health Canada authorized its use following the interim results of the Phase 2/3 double-blind placebo-controlled EPIC-HR (Evaluation of Protease Inhibition for COVID-19 in High-Risk Patients) trial demonstrating reduction in COVID-19-related hospitalization and all-cause mortality ((1,2)). Nirmatrelvir/Ritonavir was the first oral antiviral approved to treat COVID-19 in Canada. The Government of Canada assumed the role of procurement, with jurisdictions responsible for delivering the treatment to their populations. Given the unprecedented context of the fast-changing COVID-19 pandemic and the novel role of procurement, the Public Health Agency of Canada (PHAC) developed an evaluation framework in conjunction with jurisdictional stakeholders. One of the components of the framework aimed to address questions on best practices in light of the limited experience with this new therapeutic option, and to inform implementation, which this study focuses on. The evaluation of the implementation aimed to describe how the rollout of N/R took place across Canada and to identify the facilitators and challenges associated with its implementation.

### Evaluation objectives

The overarching aim of this evaluation was to answer the following questions:

1. How has N/R been administered across Canada?

2. What are the most promising strategies to deliver therapeutics in outpatient settings?

## Methods

Adopting a qualitative methodology, this study employed semi-structured group discussions as the primary method of data collection. Informed by the Donabedian framework ((3)), the Consolidated Framework for Implementation Research (CFIR) ((4)), the Reach, Effectiveness, Adoption, Implementation, and Maintenance (RE-AIM) framework (5), and behavioural science theory models ((6,7)), a structured questionnaire guided the discussions, covering key topics such as eligibility, service delivery, communication, training, and procurement experiences.

Participants were identified and recruited from various federal, provincial, and territorial (FPT) working groups using a snowball sampling technique, ensuring a diverse representation that included managers and healthcare professionals in COVID-19 therapeutics planning. Ultimately, of all jurisdictions invited, 12 provinces and territories and two federal departments participated.

One-hour interviews were conducted, and each session was recorded, transcribed, and summarized. These summaries, validated by participants, served as the foundation for descriptive and thematic analyses guided by the CFIR. The approach primarily followed a deductive method aligning with CFIR domains and constructs, while remaining open to inductive additions to the coding scheme as necessary. All factors of the CFIR were included in the initial deductive coding scheme. Each coded passage was further assigned a sentiment indicating the direction of the influencing factor (i.e., barrier or facilitator). Passages detailing the participating jurisdictions’ eligibility criteria and service delivery models were not coded but summarized.

The CFIR is a theoretical framework developed to systematically explore the intricate factors influencing the successful implementation of innovations in various organizational contexts. The CFIR’s five domains examine critical elements, including intervention characteristics, outer and inner organizational settings, individual traits, and the implementation process. Within these domains, there are 39 CFIR constructs and subconstructs, representing the evidence-based factors most likely to impact the implementation of interventions. The CFIR was used to code the data and organize emerging themes post-data collection. For the analysis, the jurisdiction responsible for the implementation was the reference unit. Nirmatrelvir/Ritonavir was coded as the innovation; the provincial, territorial, and federal healthcare organizations were coded as the inner setting; and the federal government and any other external institution as the outer setting.

The data analysis process involved initial coding through NVivo 12 ((8)) using the CFIR. This was followed by a cluster analysis to uncover patterns among the coded items based on their co-occurrence (Pearson coefficient) and, finally, matrix coding queries for constructs that were coded as positive (facilitator) or negative (challenge). Thematic analysis, inspired by Guest and Mclelan ((9)), was conducted to identify overarching themes when numerous salient themes emerged from the dataset. The findings were synthesized through a multi-step process, beginning with the identification of overarching themes through cluster analysis. Sentiments analysis was used to distinguish between facilitators and barriers, and major and minor themes were developed based on recognition of patterns within the data. The analysis included triangulation, member checking, and inter-observer reliability to bolster the credibility and transferability of the findings. Iterative rounds of analysis and refinement ensured a nuanced understanding of the complex implementation process.

### Ethics approval

The PHAC policy on research activities was followed, however, a consultation with the Research Ethics Board was not required since the implementation evaluation is within PHAC’s standard practices of assessing its programs. Consultations took place with the Privacy Management Division to ensure any personal information that might be disclosed during the evaluation was handled as per federal regulations and departmental policies.

## Results

Two main phases to the rollout of N/R were identified during the interviews: the first, at the beginning of the rollout, when the supply of N/R was limited; and the second characterized by increased and stable supply. These phases directly impacted the decision-making processes and access of N/R for Canadians.

### Eligibility criteria

At the start of the rollout, jurisdictions based their eligibility criteria for treatment with N/R on PHAC and the Canadian Agency for Drugs and Technology in Health (CADTH) guidance ((10)), the EPIC-HR trial results ((2)), the product monograph ((11)), and advice from expert advisory committees. Given the limited supply context and short lead time from drug authorization to implementation, prioritization of drug usage focused on individuals at highest risk of severe outcomes, including older, under- or unvaccinated individuals, Indigenous peoples, and those with immunosuppression, specific risk factors, or comorbidities (e.g., BMI ≥30, diabetes, lung and cardiovascular diseases). There was inter-jurisdictional variability within these criteria in regard to age thresholds for eligibility in conjunction with vaccination status and comorbidities, the number of comorbidities required for eligibility (one to three), and the comorbidities included (e.g., smoking, hypertension, chronic kidney disease).

As supply increased, most jurisdictions expanded their eligibility criteria, with a couple transitioning from defined criteria to guidance for prescribers. Changes included lowering age thresholds, inclusion of vaccinated populations, refinement of the definition of vaccination status (e.g., booster doses), and lists of comorbidities. Factors driving these changes included increased supply, new research findings (e.g., EPIC-SR trial results ((12))), evidence of a reduction in the effectiveness of neutralizing monoclonal antibodies (e.g., sotrovimab) against the new variants of concerns, and evolving epidemiology studies within the jurisdictions.

### Service delivery models

**Figure 1:**
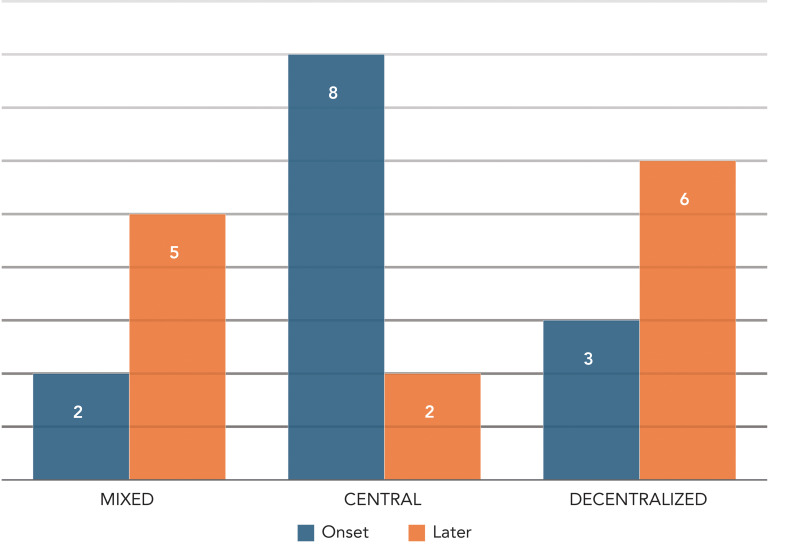
Distribution of service delivery models (SDMs) at the start of the rollout and after at least six months Distribution of service delivery models at the start of the rollout versus later (data for 13 jurisdictions). At the start of the Nirmatrelvir/Ritonavir (N/R) implementation, eight participating jurisdictions used a centralized prescription model, characterized by designated points of access and/or designated prescribers for N/R. After the initial rollout, two jurisdictions transitioned to a decentralized model, and four transitioned to a mixed model (keeping the centralized model in place for unaffiliated patients or patients that could not access their healthcare providers in a timely manner, while using a decentralized model for the majority of other patients). Three jurisdictions used a decentralized model from the beginning and continued with this approach. Two jurisdictions used a mixed model initially, with one of them transitioning to a decentralized prescription model

The service delivery models varied between jurisdictions due to differences in health system infrastructure, resulting in three main models of service delivery: centralized, decentralized, and mixed, which combines both (see **Figure 1**). Initially, most jurisdictions relied on a centralized model, enabling more oversight on distribution, which was influenced by limited supply, logistical considerations, and limited evidence to support expanded usage. However, as supply increased with time, many transitioned to a decentralized or mixed model.

Authorized prescribers varied based on jurisdictional regulations, with physicians, nurse practitioners, and pharmacists authorized to prescribe N/R. Several jurisdictions amended regulations to expand the pool of designated healthcare providers authorized to prescribe and dispense the antiviral. At the time of the data collection, five jurisdictions permitted prescriptions by pharmacists, while one allowed community nurses to prescribe in an expanded role (the numbers have since increased). The expansion of access points and authorized prescribers was noted to have increased prescribing and access to N/R by some jurisdictions.

### Thematic analysis

Based on the analysis of code relationships and their frequency of co-occurrence, seven essential themes emerged, defining the key drivers of N/R implementation in Canada (**Table 1**). The examination of barriers and facilitators to N/R implementation in Canada uncovered several key factors common across jurisdictions. These are summarized in **Table 2**, organized by CFIR domain.

**Table 1:** General themes identified from cluster analysis

Theme number	Description
Theme 1	Availability of supply, support system, and receptivity
Theme 2	Identifying needs and communicating availability
Theme 3	Coordinated efforts for efficient testing and administration
Theme 4	Overarching alignment and resolve to deliver the intervention
Theme 5	Organizational/structural synergy to facilitate intervention delivery
Theme 6	Adaptability of the implementation processes/systems
Theme 7	Concerted efforts and stakeholder engagement to prioritize high-risk populations (leveraging pre-existing systems)

**Table 2:** Consolidated Framework for Implementation Research domains and the facilitators and challenges identified (matrix coding) related to the implementation of Nirmatrelvir/Ritonavir with corresponding illustrative quotes

CFIR domain plus constructs	Facilitators Subthemes	Challenges Subthemes
Characteristic: Individual domain	**Patient and provider motivation to use the product (anecdotal)** **Quote 1:** Prescribers were happy to have access to Paxlovid^TM^, especially during the outbreak that occurred in XX from January to March 2022. **Provider capability to administer (assess and prescribe) the product** **Quote 1:** With time, as prescribers became more familiar with Paxlovid^TM^, the uptake increased.	**Perceived lack of benefits from intervention** **Quote 1:** Overall, the response and perception from healthcare practitioners (HCP) to Paxlovid^TM^ and COVID-19 therapies varied from wanting everyone to have access to concerns on the paucity of evidence.
Roles: Individual domain	**Systems in place to prioritize high risk populations – equity considerations** **Quote 1:** XX prioritized the First Nations (FN) communities in the eligibility criteria by reducing the age requirements to receive Paxlovid^TM^ and ensuring that there were access points close to the communities. **Quote 2:** The XX pathway is recommended for complex cases or unattached patients that cannot access a HCP or patients that cannot access their PCP. **Quote 3:** The XX was important in operationalizing the timely access to Paxlovid^TM^ in long-term care facilities.	N/A
Implementation: Process domain	**Flexibility of the intervention and the implementation processes to fit the context and needs** **Quote 1:** [...]. Support [from the public health organizations] was important to provide access to the vulnerable populations. As well, [...] the option of telehealth to access Paxlovid^TM^, increasing the reach and the uptake. **Development of screening and reporting tools to support the administration** **Quote 1:** The dissemination of the education sessions, order sets and protocol facilitated the rollout. The updates to the guideline were quick as were the approvals. The Office of Public Health shared information on COVID updates; Teams channels were created to post memos and documents from the various tables and committees. **Availability of alternate vs PCR testing modalities for N/R eligibility (rapid antigen tests, ID NOW, Lucera)** **Quote 1:** Furthermore, RATs have been an important tool as they are available to people in their homes and are easily accessible. This has tremendously helped the uptake of COVID-19 [treatments] by removing potential barriers in access. **Multilateral collaborative practices** **Quote 1:** The collaboration with regional medical officers of Public Health was key to the rollout. As they strategized with pharmacy leads for distribution of Paxlovid^TM^, this collaboration helped identify gaps in distribution. The rollout created new partnerships with centres to widen reach and work in collaboration to close gaps, if any, in the service delivery of Paxlovid^TM^. **Quote 2:** The excellent collaboration between pharmacists, physicians and nurses was fundamental to the success of the rollout. There was a medical advisory committee consisting of key stakeholders (pharmacists, physicians, and nurses) and there were numerous collaborative discussions to not deplete the Paxlovid^TM^ supply quickly.	**Administration processes (from screening to dispensing)** **Quote 1:** A distribution network was needed to get access to Paxlovid^TM^ in communities and facilities that may be over 2 to 3 hours away, with a courier system and taxi services to ensure that the course was started within 24 hours. **Quote 2:** There were some challenges initially as the ordering process had to be adjusted from injectable COVID-19 therapies acquired directly by the hospital for use to oral treatments, such as Paxlovid^TM^, that could be distributed and used outside of the hospital. **PCR testing** **Quote 1:** As well, the timeline and the timeliness of test results were a barrier for access as some patients may be not feel sick enough to consider testing or are tested more than 5 days post symptom onset for example, and by the time they seek care they are no longer eligible for Paxlovid^TM^. **Quote 2:** There were some challenges with ensuring that patients were tested and identified within the 5-day timeframe. There were some laboratory capacity issues creating delays with the PCR tests.
Inner setting domain	**Leveraging of pre-existing channels for dissemination of information to healthcare professionals** **Quote 1:** There were also targeted communication within networks. In effect, networks such as [healthcare professional associations] disseminated Paxlovid^TM^ communication internally through their respective newsletters to reach divisions of family practice and NPs. **Quote 2:** As well, XX and the option of telehealth to access Paxlovid^TM^, increasing the reach and the uptake. XX was able to leverage pre-existing communication pathways to disseminate information on Paxlovid^TM^ to the community, and there was also clear communication from the MOH. **Development of training and guidance materials for prescribers** **Quote 1:** [...] There were also training programs for physicians organised through presentation (in both English and French for physicians and pharmacists as the service delivery model was expanding. **Quote 2:** The guidance documents and supplementation guidance on prescribing to patients with severe kidney disease, created by the XX health renal network, was helpful as well. With time, as prescribers became more familiar with Paxlovid^TM^, the uptake increased. **Creation of new infrastructures bolstered by pre-existing pathways to facilitate implementation (e.g., IT, work processes)/Leveraging pre-existing infrastructures (IT or systems)** **Quote 1:** This network was put in place at the start of the pandemic and oversees all therapeutic recommendation approvals and the implementation processes are also discussed at the network. This has allowed for an ongoing evaluation of the process and quick responses when there were concerns with the implementation. This collaborative approach was fundamental to the rollout. **Quote 2:** The possibility to modify the regulation to allow pharmacists to prescribe was instrumental to the rollout.	**Unavailability of pre-existing IT and work infrastructures processes to respond to implementation needs (e.g., storage, workflow)** **Quote 1:** One of the main challenges to the rollout in XX was the limited resources, especially during the peak of COVID-19 infections, which coincided with the beginning of the roll out. Given the limited capacity, meeting the 5-day timeline for prescriptions was demanding, with some calls from patients frustrated because they were going to miss the treatment deadline. Providers were working overtime to ensure that patients were receiving their prescription, which also led to provider fatigue. Initially, there was no ability to follow-up on patients; now, nurses have been able to conduct day 2 and day 6 follow-ups. **Quote 2:** XX had to rely on their hospital pharmacy infrastructure to deliver the stock to community pharmacies, adding strain to resources that are not organized to perform such activities. **Quote 3:** The main deterrent from stocking Paxlovid^TM^ has been storage space as some community pharmacies do not have the space to stock high volumes given the low usage.
Innovation domain	N/A	**Perceived insufficient level of evidence on treatment effectiveness** **Quote 1:** […] the benefits of Paxlovid^TM^ were shown through a single trial carried with a select population. **Quote 2:** With limited evidence on Paxlovid^TM^, the decision-making on the eligibility and access was challenging at the onset of the rollout. **Drug-drug interactions (other products with less DDIs/easier to manage)** **Quote 1:** The response among HCPs was divided, with some being involved in the rollout and/or requesting access to Paxlovid^TM^ before its availability, while others expressed apprehension in prescribing Paxlovid^TM^ given the complexity of the drug-to-drug interactions and limited support. **Quote 2:** Initially the uptake of Paxlovid^TM^ by prescribers was slow, with some hesitancy given the complexity and the drug-to-drug interactions (DDIs).
Outer setting domain	**Multi-jurisdictional and -disciplinary collaborative practices** **Quote 1:** The willingness of the Pharmacy board to change legislation to allow pharmacists to prescribe for the treatment and prevention of COVID-19 increased access for patients and was important in rollout, especially as a proportion of the population does not have access to a HCP. The launch in the community pharmacies coupled with access to testing kits was also important in increasing access to Paxlovid^TM^ within the 5-day timeframe. **Federal procurement and availability of the intervention** **Quote 1:** Overall, XX is appreciative of the federal government role in the procurement and for providing expedited access to Paxlovid^TM^. There was support and guidance throughout the process; and the allocated stock is being used. **Quote 2:** XX recognizes the importance of PHAC taking the lead in the procurement, as obtaining supply for their population would not have been possible otherwise given the global supply shortages. The procurement was essential in providing access to their population. As well, the working groups fostered collaboration and transparency; issues were discussed as they arose, which facilitated the rollout.	**Global supply shortage context constricting communication and ability to implement** **Quote 1:** Additionally, with the limited supply at the beginning, planning the rollout and ensuring access to Paxlovid^TM^ without depleting the stock was a challenge in XX. **Quote 2:** As well, when access first expanded, some patients were not aware that Paxlovid^TM^ could be accessed through their usual providers and pharmacies; increased communication was needed to increase awareness. **Short-lead time to implement** **Quote 1:** [...] the urgency of the authorization did not follow the usual processes for clinical trials and evidence protocols. The benefits were assessed using one clinical trial and Paxlovid^TM^ was approved and distributed very quickly.

Abbreviations: DDI, drug-to-drug interactions; FN, First Nations; HCP, healthcare practitioners; MOH, Ministry of Health; NP, nurse practitioners; N/A, not applicable; N/R, Nirmatrelvir/Ritonavir; PCP, primary care provider; PCR, polymerase chain reaction; PHAC, Public Health Agency of Canada; RAT, rapid antigen tests

Adaptability to the rapidly evolving context and collaboration among stakeholders emerged as crucial facilitators, evident across individual (characteristics and roles), implementation process, and inner and outer setting domains of the CFIR. The availability of guidance documents, provided by CADTH ((10)), PHAC and expert advisory committees, at the onset of the implementation coupled with its adaptation to jurisdictional context (e.g., prioritization of equity-seeking groups and high-risk populations) for the creation of screening and reporting tools enabled a streamlined process, facilitating efficient patient prioritization, assessment, and, in some cases, follow-up. Multi-jurisdictional and interdisciplinary collaboration facilitated the development of improved processes, aided by pre-existing systems for information dissemination, access to testing and screening tools, and timely drug dispensing. Comprehensive training, continued learning, and guidance materials for prescribers contributed significantly to the intervention’s success, disseminated through various pre-existing and new channels, such as healthcare professional associations, academic institutions, and even through informal messages on a commonly used internal messaging application. The availability and admissibility of alternate testing modalities (i.e., rapid antigen and rapid molecular point-of-care tests) were instrumental in streamlining the rollout in the later stages, as well as ensuring access to N/R in remote and rural communities within the five-day eligibility window. Policies expanding the authority of pharmacists and community nurses to prescribe and administer N/R were reported as increasing uptake. Lastly, equity considerations informed the implementation of components/activities aiming to prioritize high-risk populations (e.g., long-term care residents, Indigenous populations, and racialized and marginalized individuals).

**Figure 2:**
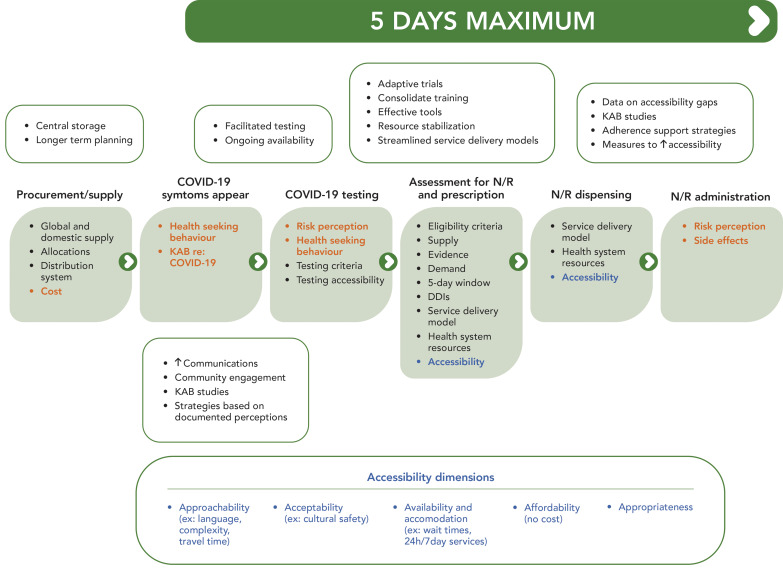
Patient trajectory to access Nirmatrelvir/Ritonavir in Canada and possible strategies (boxes outlined in green) to optimize uptake^a^ Abbreviations: DDIs, drug-drug interactions; KAB, knowledge, attitudes and behaviours; N/R, Nirmatrelvir/Ritonavir ^a^ Orange text indicates dimensions that were not explored in this evaluation; blue text indicates dimensions that were partially explored in this evaluation ((7))

Elements contributing to barriers in N/R implementation in Canada were mainly observed across the individual (characteristic), implementation process, innovation, and inner and outer setting domains of the CFIR. The perceived lack of benefits and complexity of prescribing the drug, attributed to limited evidence and numerous drug-drug interactions, influenced attitudes towards its adoption. The short lead time and global supply constraint amidst a pandemic posed challenges for the health services infrastructure, particularly in establishing necessary administration processes, IT and work infrastructures, for timely identification of eligible and best candidate patients, as well as timely delivery of the therapy. Setting up systems for identifying positive cases through PCR testing was challenging initially, but improved with widespread use of rapid antigen tests. As it relates to procurement, some jurisdictions expressed the need for enabling centralized storage capacity and federal distribution of therapeutics, alongside expanding the FPT pandemic coordination processes to include clinical discussions. The global supply constraint also influenced communication strategies and service delivery models initially, until the Canadian supply stabilized. Infrastructural limitations and insufficient human resources, including healthcare providers, further impacted the N/R rollout in Canada. A patient trajectory to access N/R in Canada along with possible strategies to streamline the process was created base on the findings from the thematic analysis (see **Figure 2**).

## Discussion

This qualitative study sought to examine the implementation of N/R within the first six months of its approval in Canada during the COVID-19 pandemic. Its aim was to identify key facilitators and challenges encountered during this implementation process and derive lessons applicable to future outpatient therapeutic implementations, thereby informing pandemic preparedness readiness in the Canadian context. While there was jurisdictional variability in eligibility criteria and service delivery models, owing to differences in health system infrastructure, the findings of this evaluation have highlighted the importance of communication, partnership and collaboration, and flexibility and adaptability of policies and implementation processes to ensure equitable access to COVID-19 treatments. In effect, jurisdictions shared knowledge and resources, from guidance documents to testing and screening mechanisms, with one another to facilitate the rollout of N/R. The context in which N/R was implemented, short lead time to implementation amidst a global shortage, along with elements directly related to the therapeutic (e.g., complexity of the therapeutics due to numerous drug-drug interactions and limited evidence, and short window of eligibility), as well as infrastructural and workforce limitations, posed challenges to its implementation.

Through our analysis, 10 themes were delineated related to facilitators and eight themes related to challenges (see Table 2). While the factors affecting uptake and barriers vary by jurisdiction based on healthcare system structure, population characteristics, and data analysis capacity, synthesizing insights from participants and considering challenges and successful approaches, strategies have been proposed to address some of the barriers uncovered in this study (Figure 2). These strategies span across the N/R supply chain from procurement through dispensing or administration to patients. Healthcare system capacity notably affected the N/R rollout, with primary care capacity being a critical factor raised by evaluation participants. While some challenges are systemic and complex, actionable strategies could include consolidating provider training, streamlining patient pathways, and ensuring continued access to rapid testing for patients eligible for treatment. Addressing cost barriers by providing free testing and treatments is crucial, especially since COVID-19 impacts are not uniformly distributed across Canadian populations ((13)). Although variations in implementation are to be expected, given the wide breadth of the population, with each jurisdiction facing some unique challenges. Furthermore, lessons learned from the implementation of N/R can help guide future therapeutics implementation efforts and inform ongoing federal planning to bolster Canada’s readiness for public health threats as Canada updates its pandemic preparedness plan ((14)). These can inform logistical planning of therapeutics implementation (e.g., testing, storage, and surveillance of distribution and dispensing), as well as the facilitation of FPT and Indigenous collaboration during the decision-making processes to ensure equitable access and distribution of therapeutics in future health emergency situations.

### Limitations

There was a high participation level, with almost all jurisdictions participating in the evaluation. The participants in the discussion session were diverse and included individuals that contributed to different capacities (i.e., healthcare professionals and managers). As such, experiences from across the country were captured, giving a broad understanding of the implementation processes of N/R. There are, however, potential limitations to the evaluation. the patient perspectives and perspectives of other more local/regional components of the healthcare system were not explored, and any information obtained was from the participants’ experiences. The potential dynamics between participants cannot be ruled as discussion sessions were held in groups for each jurisdiction. As well, although it was communicated that the results of the evaluation would be anonymized and the acquired data handled to ensure participant privacy, the potential for desirability bias remains, as the discussion sessions were led by PHAC employees. There is also the potential for interviewees’ recall bias, as the evaluation covered the experiences in the first six months of the rollout. Furthermore, while the CFIR offers great insight into the implementation processes, the complexity and scope of its 39 constructs may pose a limitation to its generalizability and consistency of its application; these limitations have been mitigated by defining the scope of the settings to ease with reproducibility.

## Conclusion

Patients’ health-seeking behaviours have been found to be influenced by their knowledge, attitudes, and beliefs ((6,7)) as well as accessibility to testing and treatment services. While this evaluation did not assess knowledge, attitudes and behaviours for patients and providers, future work in this area would further inform therapeutic implementation efforts and help understand and address some of the challenges identified in this study.

The evaluation assessed how N/R was administered in Canada in the first six months of the implementation, identifying pressure points and considerations for the initial stages of a therapeutic rollout. While jurisdictions have since modified their N/R programs, the lessons learned remain valuable for future therapeutic rollout in the context of a public health threat.
